# Flexible and high quality plant growth prediction with limited data

**DOI:** 10.3389/fpls.2022.989304

**Published:** 2022-09-12

**Authors:** Yao Meng, Mingle Xu, Sook Yoon, Yongchae Jeong, Dong Sun Park

**Affiliations:** ^1^Department of Electronics Engineering, Jeonbuk National University, Jeonbuk, South Korea; ^2^Core Research Institute of Intelligent Robots, Jeonbuk National University, Jeonbuk, South Korea; ^3^Department of Computer Engineering, Mokpo National University, Jeonnam, South Korea; ^4^Division of Electronics and Information Engineering, Jeonbuk National University, Jeonbuk, South Korea

**Keywords:** plant growth prediction, deep learning, data augmentation, T-Mixup, T-Copy-Paste, generative adversarial loss

## Abstract

Predicting plant growth is a fundamental challenge that can be employed to analyze plants and further make decisions to have healthy plants with high yields. Deep learning has recently been showing its potential to address this challenge in recent years, however, there are still two issues. First, image-based plant growth prediction is currently taken either from time series or image generation viewpoints, resulting in a flexible learning framework and clear predictions, respectively. Second, deep learning-based algorithms are notorious to require a large-scale dataset to obtain a competing performance but collecting enough data is time-consuming and expensive. To address the issues, we consider the plant growth prediction from both viewpoints with two new time-series data augmentation algorithms. To be more specific, we raise a new framework with a length-changeable time-series processing unit to generate images flexibly. A generative adversarial loss is utilized to optimize our model to obtain high-quality images. Furthermore, we first recognize three key points to perform time-series data augmentation and then put forward T-Mixup and T-Copy-Paste. T-Mixup fuses images from a different time pixel-wise while T-Copy-Paste makes new time-series images with a different background by reusing individual leaves extracted from the existing dataset. We perform our method in a public dataset and achieve superior results, such as the generated RGB images and instance masks securing an average PSNR of 27.53 and 27.62, respectively, compared to the previously best 26.55 and 26.92.

## 1. Introduction

It is estimated that one in ten people worldwide suffered from hunger and nearly one in three people lacked regular access to adequate food in 2021 according to the United Nations^1^. In addition, 149.2 million children under the age of five suffered from stunting in 2021. Hence, one goal of the United Nations is to end hunger, achieve food security and improved nutrition, and promote sustainable agriculture (Sachs et al., 2022). Simultaneously, high-quality food production has been becoming a high-level social problem in many countries, especially in developing countries mainly because agricultural development and food availability are not compatible with the distribution and changes in population in the world (Xu et al., 2021). Securing a high yield at an affordable cost is one way to mitigate this problem. To achieve this goal, analyzing plant growth under different controlled conditions is essential as they are impacted by many factors such as the supply of fertilizer and water, and further can instruct growers to take early measures when plants are not growing well. Image-based plant growth prediction has been developing in recent years due to the high availability of RGB images and the non-invasiveness of digital cameras, which can be achieved by generating high-quality future images based on previous ones. Deep learning-based methods have recently been showing great potential for image-based plant growth prediction (Somov et al., 2018; Sakurai et al., 2019; Yasrab et al., 2021), however, there are still two challenges.

First, image-based plant growth prediction is currently taken either from time-series or image-generation viewpoints, which leads to a flexible prediction framework (Sakurai et al., 2019) or more clear images (Hamamoto et al., 2020a,b; Yasrab et al., 2021), respectively. On one hand, the time-series task using long-short term memory (LSTM) (Sakurai et al., 2019) allows a changeable length of input and output, which gives more flexibility to train or test a prediction model. On the other hand, the image generation task aims to produce desired images such as high quality and high diversity (Isola et al., 2017), with which conditional generative adversarial network (GAN) loss (Goodfellow et al., 2014) can be leveraged (Hamamoto et al., 2020a; Yasrab et al., 2021) to have high quality generated images. As the advantages of time-series and image generation, we consider the plant growth prediction from both viewpoints to have clear images and a flexible framework simultaneously. Besides, plant growth prediction can be performed on two levels to get diverse information, plant-level and leaf-level. The plant level (Hamamoto et al., 2020a; Jung et al., 2022; Kim et al., 2022) requires generating RGB images, that are visually meaningful to humans, and gives the whole plant situation. In contrast, the leaf-level (Sakurai et al., 2019; Yasrab et al., 2021) demands the assignment of a leaf identity to each plant pixel and thus can be further utilized to analyze each leaf individually. However, current articles tend to perform only one level prediction (Hamamoto et al., 2020a; Yasrab et al., 2021; Jung et al., 2022; Kim et al., 2022). Diversely, we cast predicting RGB image to a regression task but instance mask as a multi-class classification task to have better plant-level and leaf-level prediction simultaneously. Table 1 gives a glimpse of related studies on the prediction content.

**Table 1:** Related study is considered from two points, the predicting content and the adopted strategy.

	**Prediction content**			**Adopted strategy**		
	**Level**	**RGB**	**IM**	**TS**	**IG**	**DA**
Sakurai et al. (2019)	Leaf	✓	✓	✓	✗	✗
Hamamoto et al. (2020a)	Plant	✓	✗	✓	✓	✗
Hamamoto et al. (2020b)^a^	Leaf	✓	✓	✓	✓	✗
Yasrab et al. (2021)	Plant	✗	✓	✗	✓	✗
Kim et al. (2022)	Plant	✓	✗	✓	✗	✗
Jung et al. (2022)	Plant	✓	✗	✓	✗	✗
Ours	Leaf	✓	✓	✓	✓	✓

Level denotes the predicting level, plant-level, or leaf-level. RGB and IM suggest predicting RGB image and instance mask. TS, IG, and DA are time-series, image generation, and data augmentation. ^a^One more input, depth information of leaves, is used in this article.

Second, a large-scale dataset is entailed for deep learning-based algorithms in the training process to obtain competitive performance, however, collecting data is time-consuming and expensive in most applications and more inconvenient for plant growth prediction as the time-series character. Although many data augmentation methods have been proposed and verified to address this challenge (DeVries and Taylor, 2017; Zhang et al., 2018; Yun et al., 2019; Xu et al., 2022), the time-series data augmentation algorithm seems underdeveloped. Since plants grow in three-dimensional space over time, three key points can be considered to do data augmentation for plant growth prediction. *Time-series* first is required in the sense that every plant or leaf should appear in its proper position or size over time. For example, plants or leaves should appear in a similar location, new leaves should be on top of old leaves, and smaller size of plants should exist in the beginning stage, instead of the latter stage. Second, *growth characteristics* and plant growth characteristics are considered in that every plant or leaf should also grow relatively freely in three-dimensional space while keeping its growth habit. Two popular non-time-series methods, Cutout (DeVries and Taylor, 2017) and Cutmix (Yun et al., 2019), conflict with this requirement in that they may split one leaf and spatially combine two leaves. Third, *useful variations* are embraced to make the trained model robust (Xu et al., 2022), such as different backgrounds, locations of leaves, and relative positions among leaves. Embracing the three points, we propose two time-series data augmentation, time-series Mixup (T-Mixup) and time-series Copy-Paste (T-Copy-Paste) based on Zhang et al. (2018), Ghiasi et al. (2021). To be more specific, T-Mixup spatially fuses two images, leading to visually no meaningful images, and thus only be leveraged to pre-train our model. T-Copy-Paste consists of two steps where clean backgrounds and desired leaves are first copied and then pasted together to form time-series images. We notice that Copy-Paste is also used as data augmentation for leaf segmentation and counting *via* combining leaves and backgrounds (Kuznichov et al., 2019), similar to ours but not for time-series data augmentation. As shown in Table 1, little related study considers the data augmentation to mitigate the limited dataset challenge in the plant growth prediction while our experimental results suggest that it significantly contributes to the performance of both RGB image and instance mask.

To summarize, our contributions are as follows:

(1) We consider the plant growth prediction from two perspectives of time series and image generation to generate good-quality images and maintain a flexible framework. Furthermore, we execute plant growth prediction at leaf-level which is more challenging and beneficial to downstream works, instead of just plant-level.
(2) We recognize three key points to perform time-series data augmentation for plant growth prediction, *time-series, growth characteristics*, and *useful variations*. Based on these points, we propose two time-series data augmentation, T-Mixup, and T-Copy-Paste, which can also be utilized for other time-series tasks.
(3) We perform our model and data augmentation in the KOMATSUNA dataset (Uchiyama et al., 2017) and achieve superior results. The generated RGB images and instance masks secured PSNR 27.53 and 27.62, compared to the previously best 26.55 and 26.92.

The remainder of this article is organized as follows. The proposed method to do plant growth prediction is instantiated in the next section, including the framework, loss function, and data augmentation method. In the experiments section, we show the implementation to train and test our model, comparison with other methods, ablation study to understand our algorithm, and flexible experiments. Finally, we conclude our study and future study in the last section.

## 2. Methods

As discussed in the introduction section, we aim to predict plant growth based on images from both time series and image generation viewpoints. Besides, we predict RGB image and leaf instance mask simultaneously to make the downstream application possible and easier. In this section, we first describe the whole framework of our method and the loss function to train the framework. Then two proposed time-series image augmentation algorithms are introduced to facilitate the limitation of the plant growth prediction dataset.

### 2.1. Framework

**Figure 1:**
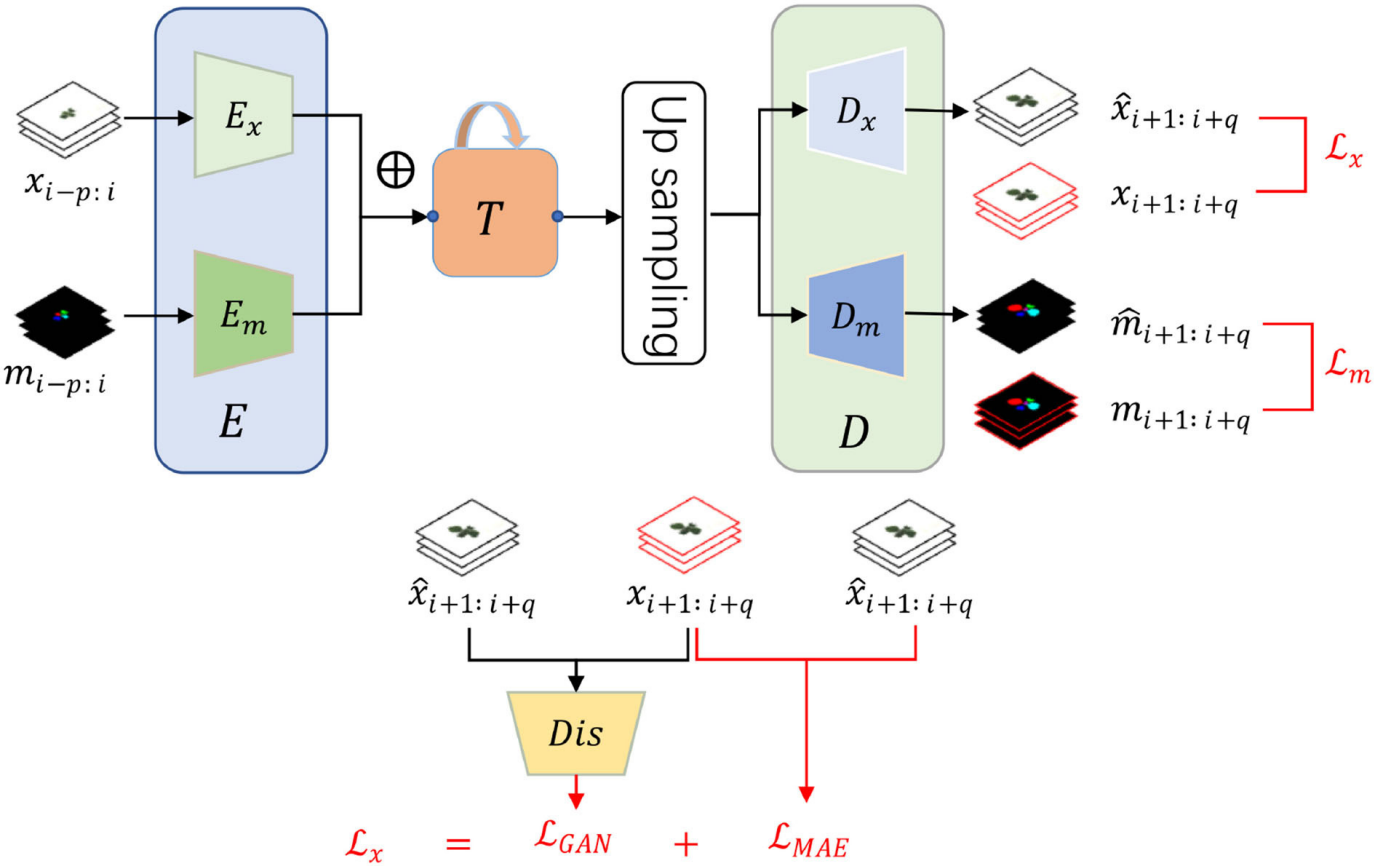
The framework and loss function of our proposed plant growth prediction model consist of three modules, input encoder *E*, output decoder *D*, and an intermediate time-series processing unit *T*. Encoder and decoder have an individual version of RGB image and instance mask because of the heterogeneity. There are two time-series inputs, historical RGB images *x*_*i*−*p* : *i*_ and instance masks *m*_*i*−*p* : *i*_ with two corresponding future predictions, RGB images ${\hat{x}}_{i+1:i+q}$ and instance masks ${\hat{m}}_{i+1:i+q}$. ⊕ represents element-wise addition. To train the networks, we design two specific loss functions $L_{x}$ and $L_{m}$ for RGB images and instance masks, respectively.

As illustrated in Figure 1, our framework consists of three main modules, encoder *E*, decoder *D*, and an intermediate time-series processing unit *T*. Functionally, the encoder is utilized to extract necessary information from the input RGB images and instance masks while the decoder aims to predict the future ones given the historical features from the time-series processing unit *T*. Differently, *T* is employed to integrate the multiple time-series features and generate multiple future features. Additionally, individual encoder and decoder for RGB images and instance masks are utilized as their heterogeneity, denoted as *E*_*x*_, *E*_*m*_, *D*_*x*_, and *D*_*m*_. Despite the heterogeneity, we assume that they are useful to predict each other as they are paired in each time step, inspired by multi-task learning (Ruder, 2017). Therefore, they are added element-wise after encoding while becoming specific before decoding. To summarize, the framework has two inputs and two outputs by which we can predict the future *q* RGB images ${\hat{x}}_{i+1:i+q}$ and instance masks ${\hat{m}}_{i+1:i+q}$ by observing the historical *p* RGB images *x*_*i*−*p* : *i*_ and instance masks *m*_*i*−*p* : *i*_. Mathematically, our framework can be formalized as:


(1) $$\left\{ \begin{array}{l} {\hat{x}}_{i+1\;:\;i+q}=D_{x}(T(E_{x}(x_{i-p\;:\;i})+E_{m}(m_{i-p\;:\;i}))),\\ {\hat{m}}_{i+1\;:\;i+q}=D_{m}(T(E_{x}(x_{i-p\;:\;i})+E_{m}(m_{i-p\;:\;i}))). \end{array}\right.$$

**Figure 2:**
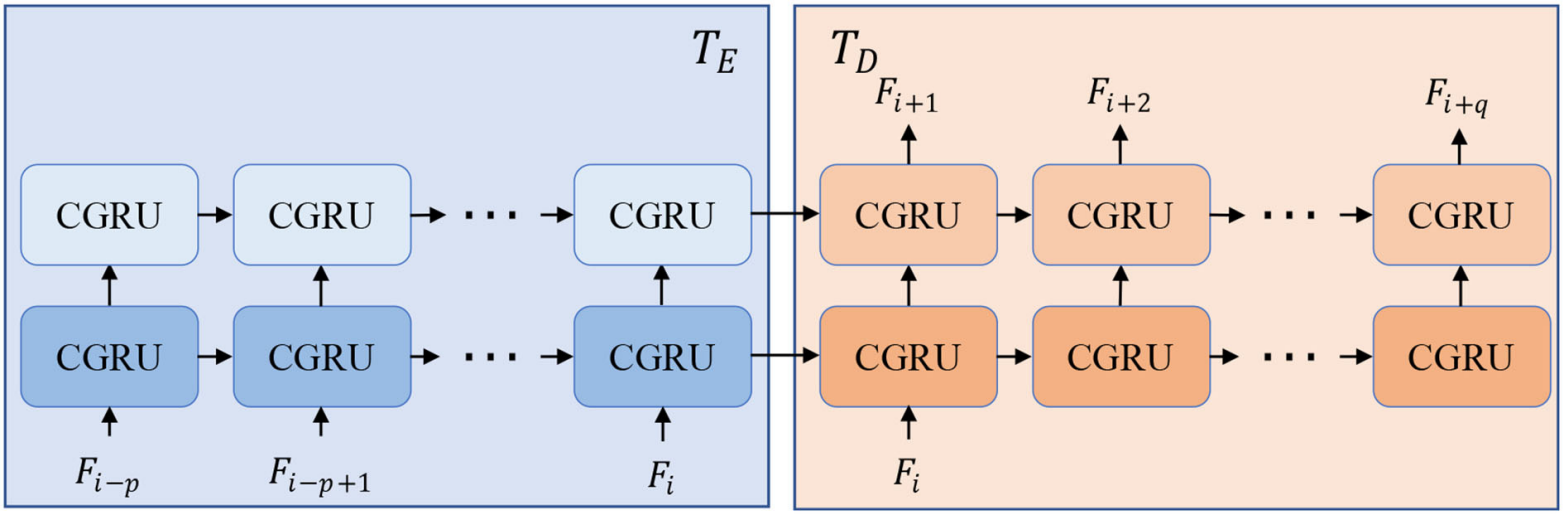
Structure of time-series processing unit *T* that is composed of a time-series encoder *T*_*E*_ and decoder *T*_*D*_. With *T*_*E*_ and *T*_*D*_, our model can predict different lengths of outputs with length-changeable inputs.

To be more specific, we employ convolution neural networks (CNN) to form the encoders and decoders because of their excellent performance and good reputations in recent years (Krizhevsky et al., 2012; He et al., 2016). In terms of the time-series processing unit, Gated Recurrent Unit (GRU) in a CNN version is borrowed because of its smaller computations than LSTM. The structure of *T* is displayed in Figure 2 and can be split into two parts, time-series encoder *T*_*E*_ and decoder *T*_*D*_. *T*_*E*_ absorbs a series of input features *F*_*i*−*p* : *i*_ with several CGRU (CNN-based GRU) cells while *T*_*D*_ sequentially predicts the future features *F*_*i*+1:*i*+*q*_ by taking the final output of the encoder. The details of each cell of CGRU can be found in the Supplementary Material. With the times-series encoder and decoder, our model is flexible to take length-changeable historical inputs and predict RGB images and instance masks with diverse time steps.

### 2.2. Loss function

As discussed in Section 1, we take the RGB images and instance masks in different ways to obtain high-quality predictions. To be more clear, we consider the RGB images from both time series and conditional image generation while thinking of predicting the instance masks as multi-class classification. Formally, two-loss functions are designed to train our model:


(2) $$\begin{array}{r} L={\text{λ}}_{x}L_{x}+{\text{λ}}_{m}L_{m}, \end{array}$$

where $L_{x}$ and $L_{m}$ are the individual loss for RGB images and instance masks, respectively. To balance the two losses, two corresponding hyper-parameters are utilized, λ_*x*_ and λ_*m*_.

First, our image loss function consists of two parts but in time-series, following paired image generation (Isola et al., 2017):


(3) $$\left\{ \begin{array}{l} L_{x}={\text{λ}}_{MAE}L_{MAE}+L_{GAN},\\ L_{MAE}=\frac{1}{q}\sum_{j=1}^{q}\|{\hat{x}}_{j}-x_{j}{\|}_{1},\\ L_{GAN}=E_{\hat{x}\sim p(gen)}\frac{1}{q}\sum_{j=1}^{q}(Dis({\hat{x}}_{j})-1{)}^{2}, \end{array}\right.$$

where $L_{MAE}$ is the image regression loss while $L_{GAN}$ aims to produce high-quality images. For image regression loss, L1 is borrowed as its resulting sharpen images as proved in (Isola et al., 2017). *p*(*real*) and *p*(*gen*) suggest the distribution of the real images and the predicted RGB images, respectively. *Dis* denotes the binary discriminator and the generator is our proposed prediction model, consisting of *E*_*x*_, *E*_*m*_, *T*, and *D*_*x*_, but without *D*_*m*_. Different from general GAN loss with only one image (Isola et al., 2017; Xu et al., 2021), our objective is for *q* time-series image generation. Simultaneously, we use the following loss function, $L_{Dis}$, to update the discriminator:


(4) $$\begin{array}{l} L_{Dis}=E_{x\sim p(real)}\frac{1}{q}\sum_{j=1}^{q}(Dis(x_{j})-1{)}^{2}\\ \;+E_{\hat{x}\sim p(gen)}\frac{1}{q}\sum_{j=1}^{q}(Dis({\hat{x}}_{j}){)}^{2}. \end{array}$$

Second, a usual multi-class classification is utilized to optimize the instance mask prediction model:


(5) $$\left\{ \begin{array}{l} L_{m}=\frac{1}{q}\sum_{j=1}^{q}-log(p(y|{\hat{m}}_{j})),\\ p(y=k|{\hat{m}}_{j})=\frac{exp({\hat{m}}_{j}^{k})}{\sum_{c}exp({\hat{m}}_{j}^{c})}, \end{array}\right.$$

where *y* is the corresponding instance label with ground truth class *k*. $p\left(y=k|\hat{m}\right)$ is the prediction score of instance masks produced by our model *D*_*m*_.

### 2.3. Data augmentation method

As mentioned before, we recognize three key points to perform time-series data augmentation for plant growth prediction, *time-series, growing character*, and *useful variations*. Based on these three points, we propose two time-series data augmentation, T-Mixup, and T-Copy-Paste.

#### 2.3.1. T-Mixup

**Figure 3:**
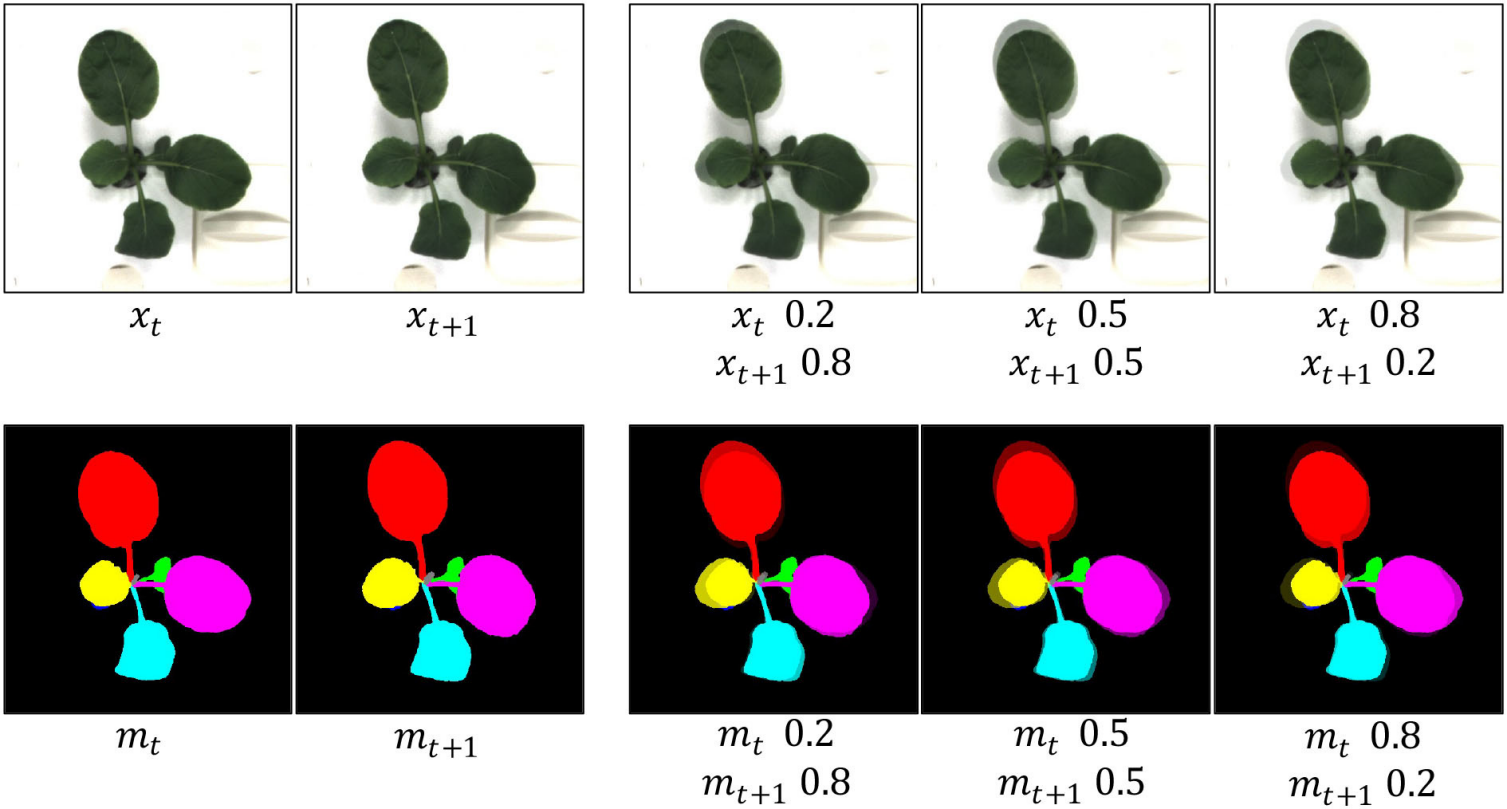
Generated examples of T-Mixup with the different merged ratios. The augmented RGB images and instance masks are not natural. Therefore, we only use T-Mixup in a pretraining process, followed by a finetuning process for all layers.

Mixup (Zhang et al., 2018) can favor linear behavior in-between training samples and keep both features of two samples. Inspired by this idea, We propose T-Mixup by spatially fusing two adjacent frames to learn the intermediate states of the same leaf between different frames. It can be formulated as:


(6) $$\left\{ \begin{array}{l} x_{new}=\text{λ}x_{i}+(1-\text{λ})x_{i+1},\\ m_{new}=\text{λ}m_{i}+(1-\text{λ})m_{i+1}, \end{array}\right.$$

where λ, a value ranging from 0 to 1, denotes the merged ratio of two frames. Although it shows superiority in many applications, it results in unnatural images for human eyes (Yun et al., 2019) as shown in Figure 3. Furthermore, we assume that unnatural images are not beneficial to image generation, though it contributes to image classification without time-series. Therefore, we apply a different strategy to utilize the T-Mixup data augmentation. Specifically, we adopt T-Mixup only to pre-train our model, followed by finetuning all layers with natural RGB images and instance masks. The ablation study in the next section proves our assumption and our strategy.

#### 2.3.2. T-Copy-Paste

**Figure 4:**
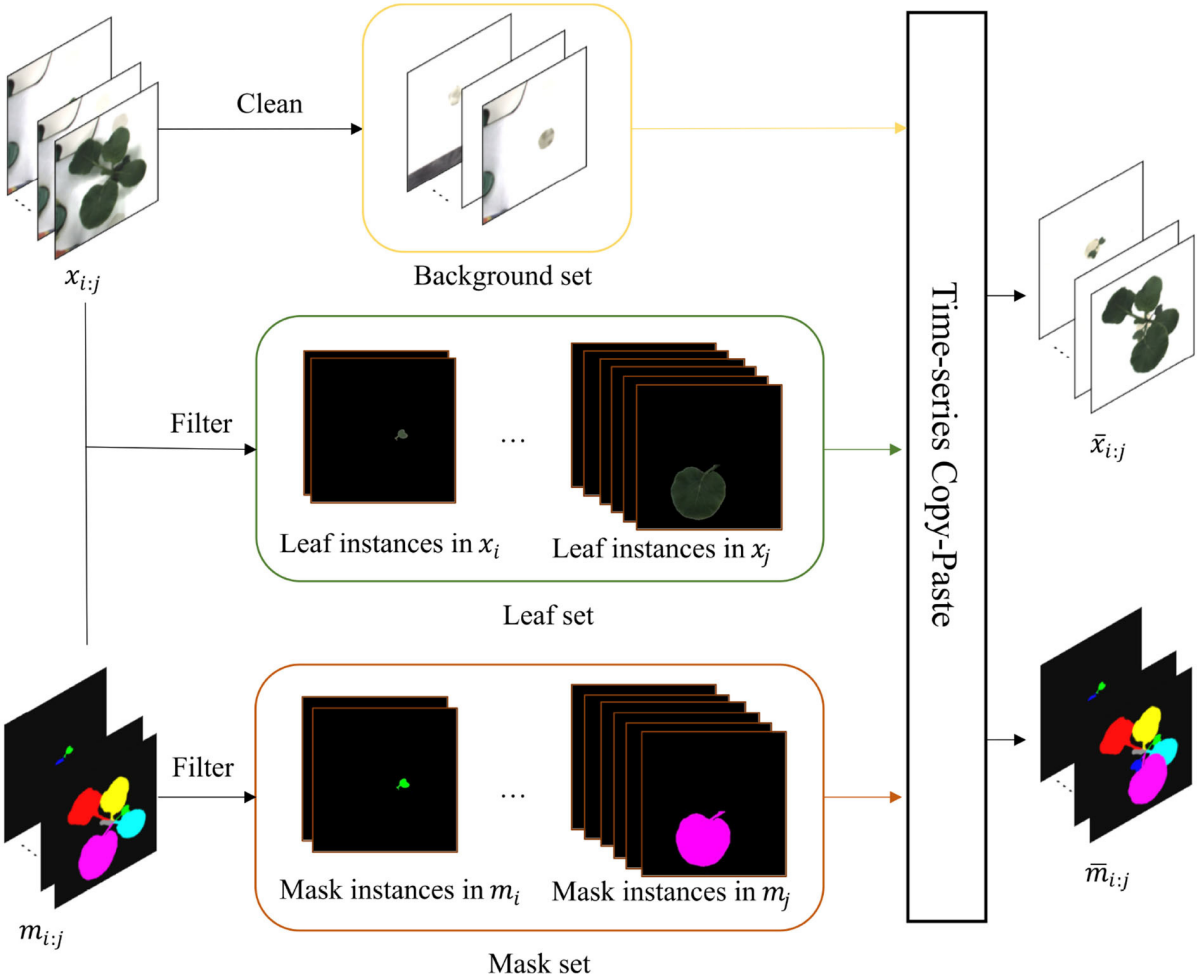
T-Copy-Paste data augmentation process. It consists of two steps: building up three sets of background, leaf, and mask, respectively from the given original dataset, and generating new RGB image and instance mask sequences by doing copy and paste of element(s) from the three sets sequentially over time. The *x*_*i* : *j*_ and *m*_*i* : *j*_ are the input sequences of RGB images and instance masks, respectively. After obtaining a clean background, suitable leaves, and their corresponding masks, we can generate new time-series images with copy and paste operation. The ${\bar{x}}_{i:j}$ and ${\bar{m}}_{i:j}$ denote generated new RGB image and instance mask sequences, respectively. Here, leaf-based data augmentation techniques can be applied together.

Copy-Paste (Ghiasi et al., 2021) is a powerful data augmentation method borrowed from the agricultural field (Kuznichov et al., 2019). However, it can not be intact to deploy in plant growth prediction due to the time-series character. To mitigate the challenge, we instead proposed a time-series copy-paste, termed T-Copy-Paste. As illustrated in Figure 4, T-Copy-Paste consists of two steps:

- Collect individual sets of background, leaf, and its corresponding mask from the existing dataset.
- Select randomly a background from the background set and sequentially paste a leaf (or leaves) chosen randomly from the leaf set and its (their) paired mask(s) to form new RGB images and instance mask images.

**Figure 5:**
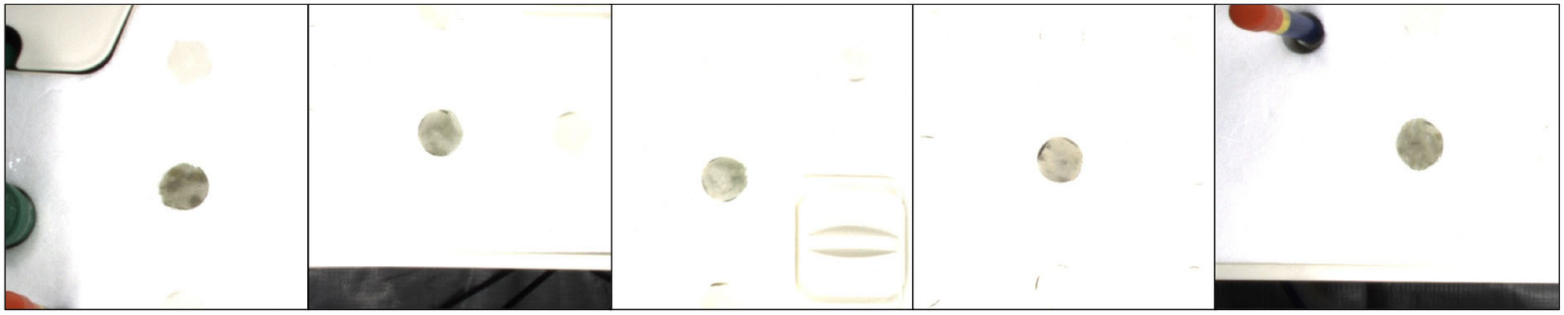
Examples of clean background images, extracted from the plants in the KOMATSUNA dataset.

To collect clean background without any leaves, we utilize an open software, GNU Image Manipulation Program with a heal-selection filter plugin (National Bureau of Statistics, 2018), that can replace manually selected areas with their surrounding pixels. Some created backgrounds are shown in Figure 5. Different from making a background set, the leaf and mask set consist of leaf instances in RGB images and their corresponding instance masks, respectively. Every leaf is in a time series and paired with its corresponding instance mask. To choose the appropriate leaves and masks for the following operations, leaves that are partially invisible or divided into some parts due to overlapping leaves have been removed because it is difficult to recover them. We use a filter process to remove the undesired RGB images or instance masks, as shown in Figure 4.

**Figure 6:**
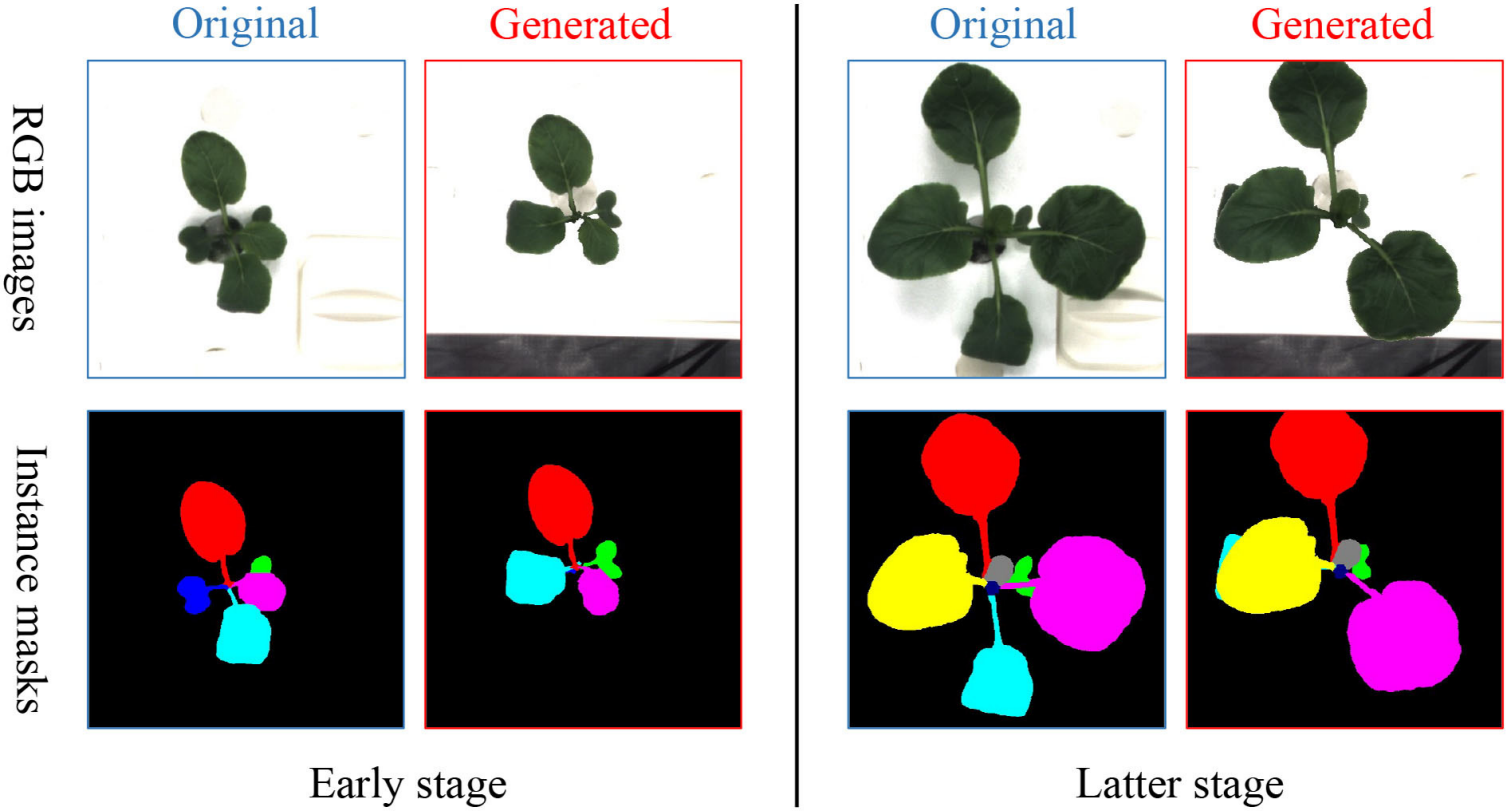
Examples of images and masks generated from T-Copy-Paste with leaf-based rotation.

After collecting the clean background, suitable leaves, and paired masks, we can produce a new set of time-series images consisting of RGB and mask that represent a plant by using the copy and paste operation. First, a background image is randomly selected and shared in time with the same plant. Then, leaf instances and their masks for the plant are selected, followed by random rotation or scale. Finally, they are copied and pasted to the chosen background to form a series of new RGB images and instance masks. The generated new RGB images and instance masks are illustrated in Figure 6.

## 3. Experiments

### 3.1. Experimental settings

#### 3.1.1. Metric

We use three evaluation metrics to assess the proposed method's performance: Dice (Eelbode et al., 2020), peak-signal-to-noise ratio (PSNR), and the structural similarity index measure (SSIM) (Hore and Ziou, 2010). Specifically, the Dice coefficient is employed to quantify the performance of image segmentation, defined as twice the overlap area of predicted and ground truth over the total number of pixels in both images. In the plant growth prediction task, the generated leaves are more important than the background and thus we borrow Dice to measure the quality of generated plant leaves. PSNR is derived from MSE but is more sensitive to image noise. SSIM is used for measuring the similarity between two images. Generally, the higher the value of Dice, PSNR, and SSIM, the better the quality of the predicted image.

#### 3.1.2. Dataset

We use KOMASTUNA (Uchiyama et al., 2017) dataset to evaluate our proposed model and data augmentation methods. The dataset contains 5 plants taken from the top and each plant consists of 60 frames acquired every 4 h in 10 days from 3 viewpoints. Besides, it also offers instance masks for each plant, in which the same label is assigned to the same leaf in all the frames and the label corresponds to the order of new leaves. In the dataset, plants have eight leaves at most, and therefore, eight is the number of classes to predict the instance mask. For the experiments, the original data are split into testing and training data at the plant level. We use 12-plant data for the training and 3-plant data for testing. Besides generic data augmentation, random rotation is utilized. The details are referred to the in Supplemental Material. Simultaneously, 40 plants in time-series are made with our proposed T-Copy-Paste data augmentation. The generic data augmentation and T-Copy-Paste are executed in an offline way while T-Mixup is in an online way.

#### 3.1.3. Implementation details

In the training process, we use the AdamW optimizer to train our model for 200 epochs with a learning rate of 0.0001. The batch size is set as 4 with two RTX 3090 GPUs (24GB memory). The dropout is used in the convolution layer of the *CGRU cell* with a dropout rate of 0.2. We execute three times and report the mean and SD for each experiment. The training processing without our proposed data augmentation costs around 7 h while spending 21 h with the proposed data augmentation as the numbers of images and instance masks increased. By default, we predict one future frame by observing three historical frames.

#### 3.1.4. Architecture details

The proposed plant growth prediction model consists of three sub-modules. First, the input encoder *E* consists of an image encoder *E*_*x*_ and mask encoder *E*_*m*_. *E*_*x*_, expecting to extract features from plant RGB images, utilizes several stacks of convolutional layers and three residual blocks, while *E*_*m*_, aiming to extract features from instance masks, adopts the same number of stacks of convolutional layers only with one residual block since the mask is simpler than images. Second, the time-series processing unit *T* leverages a convolution-Sigmoid-GroupNorm and a convolution-Tanh-GroupNorm. Third, the output decoder *D* consists of image decoder *D*_*x*_ and mask decoder *D*_*m*_. They employ two stacks of convolution-ReLU-BatchNorm. To recover the size of features after convolutional, the up-sampling operation is used which employs two up samplings with two stacks of convolution-ReLU-BatchNorm. We apply the discriminator in RGB image prediction processing to generate high-quality images and the details are referred to in Table 2. Finally, our prediction module (generator) and discriminator have about 13 and 2 million parameters.

**Table 2:** The architectural details adopted in our model.

**Network**	**Input size**	**Operation**	**Normalization**	**Active function**
*E*_*x*_	(256,256,3)	Conv7-C32-S1-P3	BN	ReLU
	(256,256,32)	Conv3-C64-S2-P1	BN	ReLU
	(128,128,64)	Conv3-C128-S2-P1	BN	ReLU
	(64,64,128)	Conv3-C256-S1-P1	BN	ReLU
	(64,64,256)		Residual block * 3	
*E*_*m*_	(256,256,9)	Conv7-C32-S1-P3	BN	ReLU
	(256,256,32)	Conv3-C64-S2-P1	BN	ReLU
	(128,128,64)	Conv3-C128-S2-P1	BN	ReLU
	(64,64,128)	Conv3-C256-S1-P1	BN	ReLU
	(64,64,256)		Residual block * 1	
*T*	(64,64,256)	Conv3-C256-S1-P1	GN	Sigmoid
	(64,64,256)	Conv3-C256-S1-P1	GN	Tanh
	(64,64,256)	Conv3-C256-S1-P1	GN	Sigmoid
	(64,64,256)	Conv3-C256-S1-P1	GN	Tanh
*Up sampling*	(64,64,256)	Scale2		
	(128,128,256)	Conv3-C128-S1-P1	BN	ReLU
	(128,128,128)	Scale2		
	(256,256,128)	Conv3-C64-S1-P1	BN	ReLU
*D*_*x*_	(256,256,64)	Conv3-C32-S1-P3	BN	ReLU
	(256,256,32)	Conv3-C3-S2-P1	BN	ReLU
*D*_*m*_	(256,256,64)	Conv3-C32-S1-P1	BN	ReLU
	(256,256,32)	Conv3-C9-S1-P1	BN	ReLU
*Dis*	(256,256,3)	Conv4-C64-S2-P1	InstNorm	LeakyReLU
	(128,128,64)	Conv4-C128-S2-P1	InstNorm	LeakyReLU
	(64,64,128)	Conv4-C256-S2-P1	InstNorm	LeakyReLU
	(32,32,256)	Conv4-C512-S1-P1	InstNorm	LeakyReLU
	(31,31,256)	Conv4-C1-S1-P1	InstNorm	Sigmoid

The input size is height, width, and channel. In the operation, Conv*k* is a convolution layer with kernel size as k. C*k*, S*k*, and P*k* denote the number of channels, stride, and padding, respectively. Scale2 means the up-sampling factor is 2. Batch normalization (BN), Group Normalization (GN), and Instance normalization (InstNorm) (Wu and He, 2018) are used. We utilize three residual blocks (He et al., 2016) to extract necessary information from the image while fewer residual block is used in the mask encoder as the mask is much simpler than the image.

### 3.2. Comparisons with other methods

In this subsection, we compare our method to the related articles, ConvLSTM (Sakurai et al., 2019), FutureGAN (Yasrab et al., 2021), STN-LSTM (Jung et al., 2022), and STN-STPD (Kim et al., 2022). The main characters of the articles refer to Table 1. For ConvLSTM, we rewrite the model and randomly train the model three times, and then report the mean performance. For other three articles, we directly borrow the evaluations from their articles as their codes are not public and executing details are not enough to reproduce. Similarly, direct comparison to Hamamoto et al. (2020a,b) is somehow hard, though they are more related to our method. Furthermore, depth information is required for the methods, and therefore, we do not compare with them. Besides, two video prediction methods, MC-Net (Villegas et al., 2017a) and HP-Net (Villegas et al., 2017b), are compared since video prediction is similar to plant growth prediction. The performances of the two articles are borrowed from Kim et al. (2022). For our method, we train our model three times and give the mean evaluations while the variance can be referred in the following subsections. All experiments are executed in the same dataset, KOMASTUNA (Uchiyama et al., 2017). The comparison results are displayed in Table 3.

**Table 3:** Performance comparisons with other methods.

**Method**	***I*_*psnr*_**	***I*_*ssim*_**	***I*_*dice*_**	***M*_*psnr*_**	***M*_*ssim*_**	***M*_*dice*_**
HP-Net^+^ (Villegas et al., 2017b)	24.66	0.89	-	-	-	-
MC-Net^+^ (Villegas et al., 2017a)	25.02	0.90	-	-	-	-
ConvLSTM^*^ (Sakurai et al., 2019)	24.54	0.89	90.24	26.92	0.986	90.25
FutureGAN (Yasrab et al., 2021)	-	-	-	23.20	0.959	-
STN-LSTM (Jung et al., 2022)	25.95	0.90	-	-	-	-
STN-STPD (Kim et al., 2022)	26.55	0.91	-	-	-	-
Ours	27.53	0.92	91.88	27.62	0.990	91.88

Red font shows the best performance. + suggests that the performances are taken from (Kim et al., 2022) while ^*^ denotes that we rewrite and reproduce their code, and then show their average performance after three random training and testing processes. Otherwise, we borrow the evaluations from the original papers. Our method is randomly trained three times and the mean performance is reported while the variance is given in the following subsections.

From the table, we observe that the performances are gradually improved in recent years. In terms of predicting RGB images alone, shape information introduced in STN-LSTM (Jung et al., 2022) and STN-STPD (Kim et al., 2022) essentially improves the quality. In contrast, only using an instance mask may not be a good choice because of the poor mask PSNR in FutureGAN (Yasrab et al., 2021). We guess that RGB images have extra beneficial signals to predict instance masks. Finally, our method significantly surpasses the previous method by a clear margin on all evaluation metrics. In the following subsection, we analyze the reasons why our method contributes and the effectiveness of each module.

### 3.3. Ablation study

#### 3.3.1. Hyperparameter

**Figure 7:**
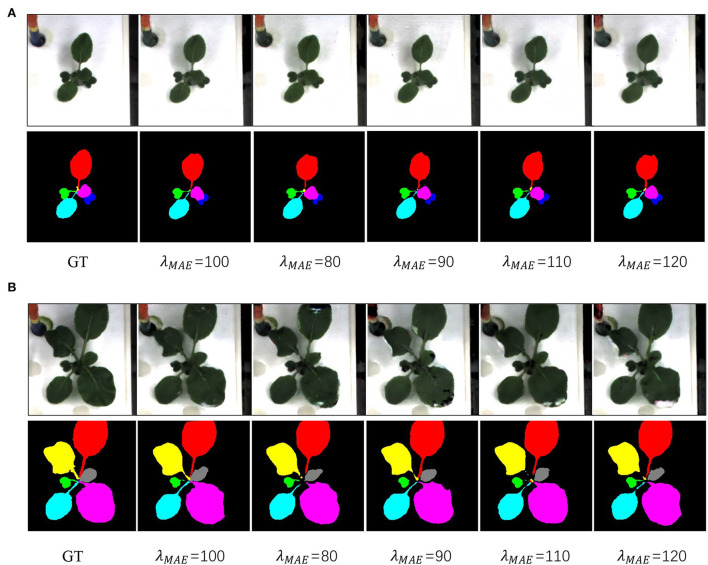
Qualitative results of different λ_*MAE*_. Panels **(A,B)** are examples from the early stage, and latter stage, respectively. GT denotes the ground truth.

In this subsection, we analyze the impacts of hyperparameters. For this hyperparameter ablation study, the proposed data augmentation methods are not used. First, λ_*MAE*_ is adopted to balance the MAE loss and adversarial loss when generating the plant RGB images. Table 4 gives the performance and Figure 7 gives a visual comparison. From the table, the performances become better and then worse when λ_*MAE*_ gradually varies from 80 to 120. Especially, the model with λ_*MAE*_ = 100 achieves the best average *I*_*psnr*_ 24.94, *M*_*psnr*_ 27.02, and Dice 90.63. Visual comparison gives similar evidence that the generated RGB images have less noise and more details with λ_*MAE*_ = 100. For example, the biggest leaf in the latter stage in Figure 7 is better with the less missing part when λ_*MAE*_ equals 100, as well as the instance mask with a better shape.

**Table 4:** Ablation study of λ_*MAE*_ in $L_{x}$ loss function for plant RGB image generation.

**λ_*MAE*_**	***I*_*psnr*_**	***I*_*dice*_**	***M*_*psnr*_**	***M*_*dice*_**
80	24.48 ±±0.07	90.07 ± 0.12	26.85 ± 0.03	90.07 ± 0.12
90	24.60 ± 0.09	90.20 ± 0.10	26.88 ± 0.02	90.20 ± 0.10
100	24.94 ± 0.03	90.63 ± 0.31	27.02 ± 0.03	90.63 ± 0.31
110	24.65 ± 0.04	90.23 ± 0.15	26.95 ± 0.06	90.23 ± 0.15
120	24.48 ± 0.10	90.10 ± 0.00	26.92 ± 0.03	90.10 ± 0.00

The red font shows the best average performance.

Second, we aim to get better qualities for both RGB image and instance mask *via* changing λ_*x*_ and λ_*m*_. The ablation results are given in Table 5. The performance becomes better when RGB images are emphasized (λ_*x*_ is larger than λ_*m*_). We argue that producing better RGB images is harder than instance masks as the RGB images have more details in a regression task.

**Table 5:** Ablation study of λ_*x*_ and λ_*m*_ in L loss function.

**λ_*x*_**	**λ_*m*_**	***I*_*psnr*_**	***I*_*dice*_**	***M*_*psnr*_**	***M*_*dice*_**
1.0	1.0	24.86 ± 0.18	90.29 ± 0.12	26.94 ± 0.02	90.29 ± 0.12
1.5	1.0	24.94 ± 0.03	90.63 ± 0.31	27.02 ± 0.03	90.63 ± 0.31
1.0	1.5	24.74 ± 0.18	90.32 ± 0.07	26.95 ± 0.03	90.32 ± 0.07
1.5	1.5	24.69 ± 0.13	90.23 ± 0.06	26.93 ± 0.02	90.27 ± 0.12

The red font shows the best average performance.

#### 3.3.2. Data augmentation

In this subsection, we analyze the impact of the proposed T-Copy-Past and T-Mixup data augmentation. The baseline employs basic data augmentation and random rotation, with which more details refer to the Supplementary Material. The experimental results are displayed in Table 6. Compared to the baseline, T-Mixup alone results in slightly worse performance for RGB images, such as the average *I*_*psnr*_ varying from 24.94 to 24.86. In contrast, the finetuning strategy is beneficial to the performance, which suggests that plant growth is different from generic image classification and needs natural images to train the prediction model. Compared to T-Mixup, T-Copy-Paste contributes more to all performances. For example, it alone takes a 2.14 improvement of PSNR of RGB images than the baseline. The combination of finetuning of T-Mixup and T-Copy-Paste leads to the largest increase, which implies that a limited dataset is one challenge to have a better plant growth prediction model and our time-series data augmentation is one effective method.

**Table 6:** Ablation study of data augmentation.

**T-Mixup**	**T-Copy-Paste**	***I*_*psnr*_**	***I*_*ssim*_**	***I*_*dice*_**	***M*_*psnr*_**	***M*_*ssim*_**	***M*_*dice*_**
✗	✗	24.94 ± 0.03	0.89 ± 0.01	90.63 ± 0.31	27.02 ± 0.03	0.99 ± 0.00	90.63 ± 0.31
✓	✗	24.86 ± 0.09	0.88 ± 0.01	90.81 ± 0.12	26.99 ± 0.06	0.98 ± 0.00	90.81 ± 0.12
Finetune	✗	24.94 ± 0.09	0.89 ± 0.01	90.84 ± 0.29	27.09 ± 0.11	0.99 ± 0.00	90.84 ± 0.29
✗	✓	27.08 ± 0.07	0.91 ± 0.00	91.21 ± 0.42	27.38 ± 0.02	0.99 ± 0.00	91.21 ± 0.42
✓	✓	27.43 ± 0.02	0.92 ± 0.00	91.58 ± 0.21	27.26 ± 0.06	0.99 ± 0.00	91.58 ± 0.21
Finetune	✓	27.53 ± 0.04	0.92 ± 0.00	91.88 ± 0.48	27.62 ± 0.03	0.99 ± 0.00	91.88 ± 0.48

Because of the unnatural images, T-Mixup is first utilized to pretrain our model and then finetuned with all layers. The red font shows the best average performance.

#### 3.3.3. Modules in our algorithm

**Figure 8:**
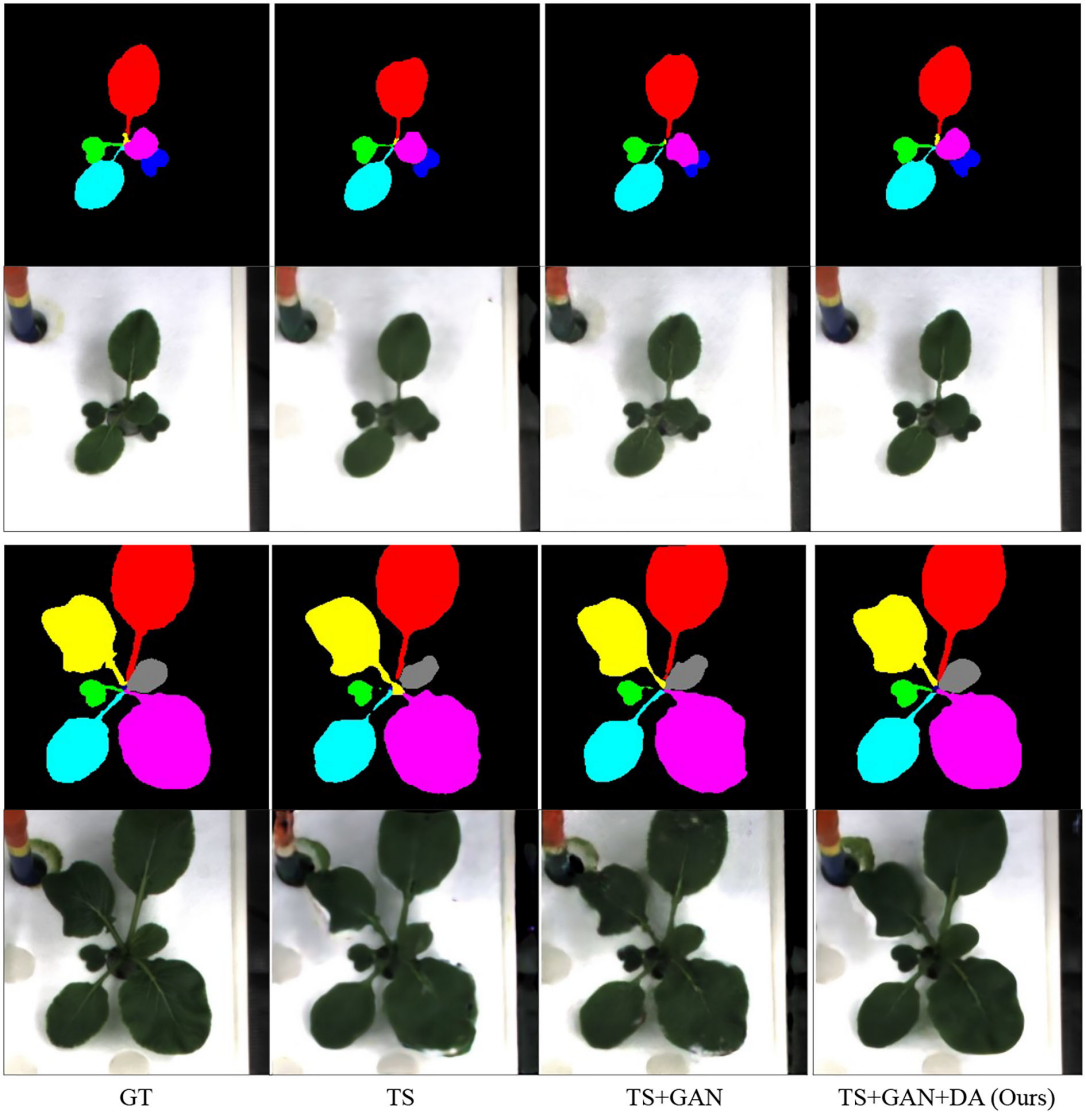
Ablation study of our algorithm. GT denotes the ground truth. TS and DA suggest time-series and our proposed time-series data augmentation.

Finally, we aim to distinguish the three main contributions in our paper, time-series, GAN, and data augmentation. The evaluation is given in Table 7 and the visual comparison is displayed in Figure 8. GAN can slightly improve the quality of RGB images and the instance masks. More interestingly, data augmentation leads to a huge improvement.

**Table 7:** Ablation study of our algorithm with three main contributions, time-series (TS), GAN, and the proposed time-series data augmentation (DA).

	***I*_*psnr*_**	***I*_*ssim*_**	***I*_*dice*_**	***M*_*psnr*_**	***M*_*ssim*_**	***M*_*dice*_**
TS	24.54 ± 0.03	0.89 ± 0.00	90.24 ± 0.05	26.92 ± 0.02	0.99 ± 0.00	90.25 ± 0.05
TS + GAN	24.94 ± 0.03	0.89 ± 0.01	90.63 ± 0.31	27.02 ± 0.03	0.99 ± 0.00	90.63 ± 0.31
TS + GAN + DA (Ours)	27.53 ± 0.04	0.92 ± 0.00	91.88 ± 0.48	27.62 ± 0.03	0.99 ± 0.00	91.88 ± 0.48

### 3.4. Flexible plant growth prediction

As discussed in Section 1, we aim to achieve flexible plant growth prediction. In this case, we train our model to predict one future frame given three historical three frames, but we can test our model in a different case. By default, our model is tested in the same way (3to1), but we can also predict two future frames given three frames (3to2) without retraining the model, as well as in the 2to1 case. The testing performance in a different case is given in Table 8. The table suggests that more history benefits better performance and predicting more future frames is harder.

**Table 8:** Testing result of flexible plant prediction model.

	***I*_*psnr*_**	***I*_*ssim*_**	***I*_*dice*_**	***M*_*psnr*_**	***M*_*ssim*_**	***M*_*dice*_**
2to1	27.20 ± 0.01	0.92 ± 0.00	90.54 ± 0.05	27.35 ± 0.01	0.99 ± 0.00	90.54 ± 0.05
3to1	27.53 ± 0.04	0.92 ± 0.00	91.88 ± 0.48	27.62 ± 0.03	0.99 ± 0.00	91.88 ± 0.48
3to2	25.41 ± 0.02	0.92 ± 0.01	87.67 ± 0.50	26.66 ± 0.01	0.99 ± 0.00	87.67 ± 0.50

atob means predicting *b* frames given *a* frames.

## 4. Conclusion

In this article, we considered the plant growth prediction from both time-series and image generation viewpoints to produce clear RGB images with a flexible framework. RGB images and instance masks of the leaf are predicted simultaneously, which suggests that our prediction is at leaf-level, instead of plant-level. With our model, we can flexibly predict different numbers of frames given diverse historical frames after training one specific model, such as predicting one frame given three input frames. Furthermore, we propose two time-series data augmentation, T-Mixup and T-Copy-Paste, to mitigate the limited dataset. Compared to the generic data augmentation such as rotation, the proposed T-Copy-Paste introduces specific variations for plant growth prediction, e.g., the spatial relations among leaves and the background. T-Mixup is related to the temporary information during plant growth and is only used to pretrain a model since the augmented images are not natural visually. The experimental results suggest that our method outperforms the current methods with a clear margin. To the best of our knowledge, we are the first ones to consider data augmentation for plant growth prediction. Especially, we believe that our data augmentation method, giving a bigger improvement than GAN, highlights the challenge of the limited dataset in plant growth prediction. In the future, we would like to validate our model in other possible datasets.

## Data availability statement

Publicly available datasets were analyzed in this study. This data can be found here: https://ieeexplore.ieee.org/document/8265449.

## Author contributions

YM conceived the idea, designed the algorithm, conducted all of the experiments, and wrote the manuscript. MX participated in the algorithm discussion and revised the manuscript. SY supervised the project and the overall improvement of the manuscript. YJ improved the manuscript. DP conceptualized the article, supervised the project, and got funding. All authors read and approved the manuscript.

## Funding

This research was supported by Basic Science Research Program through the National Research Foundation of Korea (NRF) funded by the Ministry of Education (No. 2019R1A6A1A09031717). This study was supported by the National Research Foundation of Korea (NRF) grant funded by the Korean government (MSIT) (NRF-2021R1A2C1012174), the Korea Institute of Planning and Evaluation for Technology in Food, Agriculture and Forestry (IPET) and Korea Smart Farm Foundation (KosFarm) through the Smart Farm Innovation Technology Development Program, funded by the Ministry of Agriculture, Food and Rural Affairs (MAFRA) and Ministry of Science and ICT (MSIT), and Rural Development Administration (RDA) (421027-04).

## Conflict of interest

The authors declare that the research was conducted in the absence of any commercial or financial relationships that could be construed as a potential conflict of interest.

## Publisher's note

All claims expressed in this article are solely those of the authors and do not necessarily represent those of their affiliated organizations, or those of the publisher, the editors and the reviewers. Any product that may be evaluated in this article, or claim that may be made by its manufacturer, is not guaranteed or endorsed by the publisher.

## Footnotes

^1^ https://sdgs.un.org/goals/goal2
