# Supplementary material for: Flexible and high quality plant growth prediction with limited data
Source: Front Plant Sci. 2022 Sep 12;13:989304. doi: 10.3389/fpls.2022.989304 (PMC9511019; doi:10.3389/fpls.2022.989304)
Supplement: Supplementary file 1 [file Data_Sheet_1.pdf]

Flexible and High Quality Plant Growth Prediction with Limited Data

0.1 Details of CGRU

The flowchart of convolution-based Gated Recurrent Unit (CGRU) is illustrated in Figure S1.

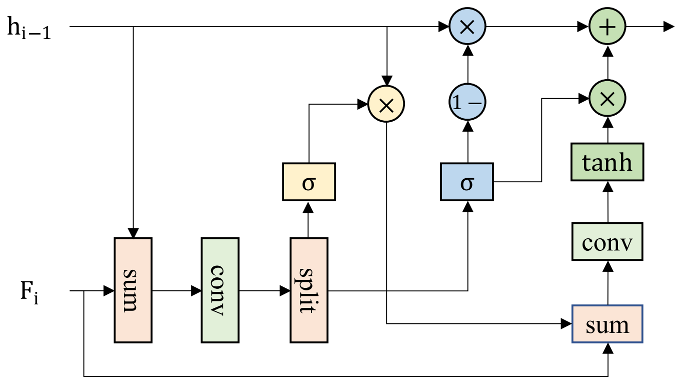

Figure S1. Flowchart of CGRU cell. It mainly consists of a reset gate and a update gate where Conv denotes a 1D-convolutional kernel to capture dependencies, while Sum denotes add operation. In this paper, we use the add operation instead of concatenation for decreasing parameters. Besides, dropout is used in the convolutional layer.

0.2 Baseline with generic data augmentation

Before performing our proposed data augmentation strategy, we conduct preliminary experiments with generic data augmentation methods. By analyzing the training and testing datasets, the orientation is one of the main variations. Hence we randomly rotate the images on the training dataset with a rotation angle ranging from -90 to 90. Simultaneously, the widely used random crop is not applied as this operation results in the missing part of leaves that are essential to predict plant growth. Under the random rotation, the times to use it may play a role. Therefore, we execute different times and the results are shown in Table S1. From the table, we can see that three times of data augmentation are not sufficient, especially in mask prediction. We also find the generated masks with massive noises. Five-times achieves the best average performance while more-times does not leads to a better performance. We argue that the model may suffer from the notorious overfitting problem the useful variation from random rotation is limited. To reduce the training time, our main work just utilize three times of generic data augmentation.

Table S1. Performances according to the number of times that the data augmentation using random rotation is applied. The I_* and M_* denote generated plant RGB image and mask, respectively. Red fonts are the best results for each evaluation metric. We execute three times and report the mean and standard deviation.

Times	I_{psnr}	I_{ssim}	I_{dice}	M_{psnr}	M_{ssim}	M_{dice}
3	24.49±0.01	0.88±0.01	85.78±0.60	24.53±0.12	0.98±0.00	85.78±0.60
4	24.62±0.06	0.89±0.01	90.28±0.09	26.88±0.04	0.99±0.00	90.28±0.09
5	24.94±0.03	0.89±0.01	90.63±0.31	27.02±0.03	0.99±0.00	90.63±0.31
6	24.72±0.05	0.89±0.01	90.03±0.06	26.88±0.07	0.99±0.00	90.03±0.06
7	24.36±0.59	0.88±0.00	89.97±0.14	26.82±0.09	0.98±0.00	90.00±0.10
8	24.34±0.04	0.88±0.00	89.91±0.25	26.81±0.09	0.98±0.00	89.99±0.12
